# Microbes for lung cancer detection: feasibility and limitations

**DOI:** 10.3389/fonc.2024.1361879

**Published:** 2024-05-08

**Authors:** Sirui Zhou, Weijian Zhu, Hehua Guo, Yalan Nie, Jiazheng Sun, Ping Liu, Yulan Zeng

**Affiliations:** ^1^ Department of Respiration, Liyuan Hospital, Tongji Medical College, Huazhong University of Science and Technology, Wuhan, China; ^2^ Department of Orthopedics, Liyuan Hospital, Tongji Medical College, Huazhong University of Science and Technology, Wuhan, China

**Keywords:** lung cancer, microbes, detection, diagnosis, biomarker

## Abstract

As the second most common cancer in the world, the development of lung cancer is closely related to factors such as heredity, environmental exposure, and lung microenvironment, etc. Early screening and diagnosis of lung cancer can be helpful for the treatment of patients. Currently, CT screening and histopathologic biopsy are widely used in the clinical detection of lung cancer, but they have many disadvantages such as false positives and invasive operations. Microbes are another genome of the human body, which has recently been shown to be closely related to chronic inflammatory, metabolic processes in the host. At the same time, they are important players in cancer development, progression, treatment, and prognosis. The use of microbes for cancer therapy has been extensively studied, however, the diagnostic role of microbes is still unclear. This review aims to summarize recent research on using microbes for lung cancer detection and present the current shortcomings of microbes in collection and detection. Finally, it also looks ahead to the clinical benefits that may accrue to patients in the future about screening and early detection.

## 1 Introduction

Influenced by smoking and environmental factors, lung cancer is a disease with a high incidence worldwide. Small-cell lung cancer (SCLC) and non-small cell lung cancer (NSCLC) are the two histological subtypes of lung cancer. SCLC accounts for 15% of lung cancers and is usually caused by smoking. At the same time, NSCLC is divided into squamous (LUSC), adenocarcinomas (LUAD), large cell carcinomas, and bronchial carcinoid cancers, of which LUAD is the most common cancer and is usually seen in non-smoking women. Due to the characteristics of insidious disease onset and easy invasion in the early stage, the five-year survival rate of lung cancer patients is only 19.8% (1). Although the emergence of immunotherapy has provided a chance of survival for patients with advanced disease, 60% of patients do not have immunotherapy driver genes, and even the immune response rate of patients with NSCLC is only 15-25% (2).In addition, some patients undergo immunotherapy for a certain period and develop immune escape, while others discontinue treatment due to severe immune-related adverse effects (3). If the disease is diagnosed early, the five-year survival rate of lung cancer patients can be increased to 59% (4).

Currently, lung cancer detection includes asymptomatic screening and diagnostic evaluation. The National Comprehensive Cancer Network (NCCN) has shown that lung cancer mortality can be reduced by a relative 20% with low-dose CT (LDCT) chest screening in high-risk populations (5). However, LDCT radiation exposure induces cancer, and false positives on LDCT images are high, with 24.2% of screened individuals still requiring further testing (6). For another, the definitive diagnosis of lung cancer is still based on invasive biopsy histopathologic testing. Still, biopsy has disadvantages such as high price, susceptibility to complications, and the need for sufficient diseased lung tissue (7). Therefore, we are eager to find potential markers that can help in the specific detection of lung cancer patients. Since tumor cells are transformed from normal cells, biomarkers are an objective assessment for the early detection of tumors. Some circulating antigens such as CYFRA21-1, CEA, NSE, and SCC-Ag have been used as markers for the clinical detection of lung cancer (7, 8) (**Table 1**).

**Table 1:** Current detection modalities for lung cancer and their characteristics.

Lung cancer detection methods	Reference	Advantages	Disadvantages
LDCT	(5)	1. 20% reduction in mortality in high-risk groups 2. Short time-consuming	1. Radiation exposure 2. False positives
Tissue biopsies	(9)	1. The most reliable method at this time	1. Invasive and time-consuming 2. Susceptibility to complications 3. Need for sufficient diseased lung tissue
Blood circulating antigens	(7, 8)	1. An objective assessment 2. Safe and fast 3. Evaluation of metastasis and recurrence	1. Low specificity 2. Need for combination with other tumor markers
Microbes	(10, 11)	1. Easy collection and less invasive 2. Repeatable collection 3. Possibility of dynamic observation of the disease process	1. Lack of standard programs 2. Susceptible to contamination

The microbiota is a community of all microbial species, including bacteria, fungi, viruses, archaea, and protozoa. Depending on their preferences for nourishment and oxygen, different microbes are often found on human body surfaces that come into touch with the external environment, including the skin, mouth, respiratory system, gastrointestinal tract, and urine tract (4). The human microbiota begins to colonize at birth. It is closely related to the maternal microbiota, how the infant is born, how he or she is fed, and the environment in which he or she survives. These microbes remain relatively stable in the body as adults through a complex symbiotic relationship with the host (12). The host can provide the environment for the microbes to metabolize nutrients. Conversely, the microbes can regulate the host’s metabolism, nutritional response, and immune function and provide the host with vitamins and trace elements (13). With the emergence of next-generation sequencing (NGS) in recent years, microbes have gained widespread attention in the development of cancer and its treatment. However, there are still many difficulties in their use for early prevention and detection. This review focuses on the possible mechanisms and research progress in using microbes for lung cancer detection and discusses the deficiencies in sample collection and testing, which provide new ideas for the precise detection of lung cancer.

## 2 Current status of microbes used for cancer detection

The use of microbes to aid in cancer detection is well-founded, with studies showing that 15.4-17.8 percent of cancers are associated with microbial infections (14). The International Agency for Research on Cancer (IARC) has identified 11 microbes as class I carcinogens, including bacteria, viruses, and parasites, such as *Helicobacter pylori (Hp)*, *Hepatitis B virus*, *HPV*, *EBV*, and *Schistosoma chinensis*, among others (15). Since the largest tribe of microbiota in the body is the gut microbiota, their association with gastrointestinal tumors has been the most studied. In the stomach, the oncoprotein cytotoxin-associated gene A (CagA) and vacuolar toxin A (VacA) produced by *Hp* increase the production of inflammatory cytokines and immune cells, as well as the methylation of tumor suppressor genes CpG islands, which ultimately lead to gastric adenocarcinoma (16). The liver has a rich circulatory system. *Hepatitis B and C viruses*, *aflatoxin B1*, and gastrointestinal flora can enter the circulation and have a major impact on the liver through the hepatic portal system (17). In addition, several studies have demonstrated that host microbes, such as *Fusobacterium nucleatum*, *Escherichia coli*, and *Bacteroides fragilis*, influence colorectal carcinogenesis by inducing intestinal inflammation (18, 19).

## 3 Possibility of microbes for lung cancer detection

**Figure 1:**
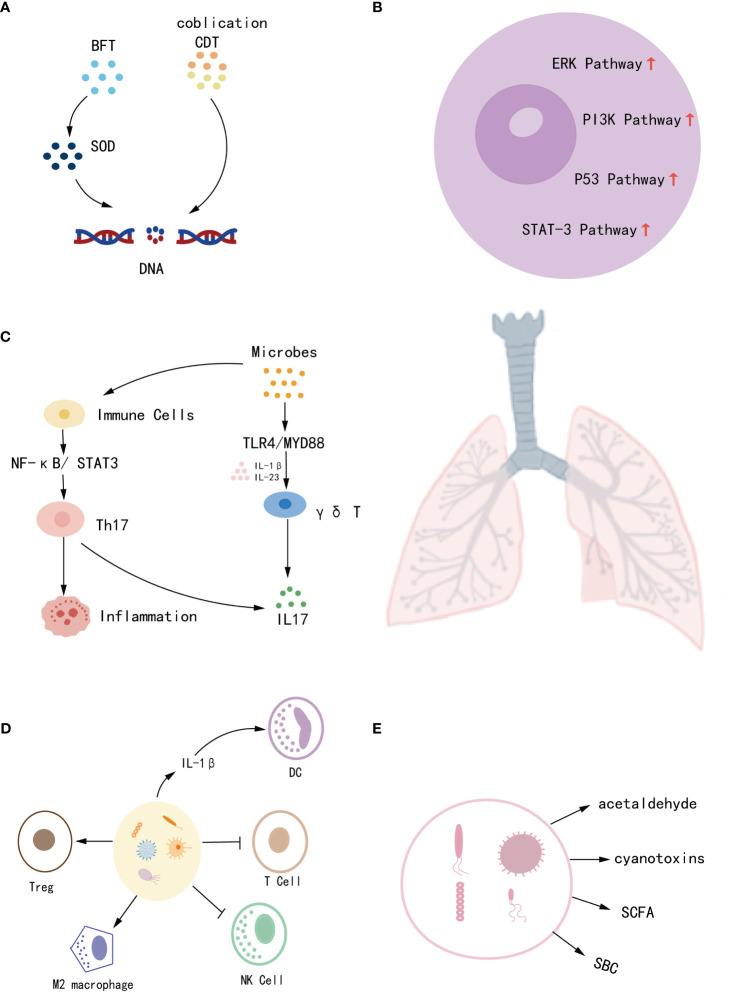
The mechanisms by which microbes contribute to the development of lung cancer include **(A)** disruption of host genes, **(B)** induction of chronic inflammation, **(C)** activation of oncogenic pathways, **(D)** influence on immune response, and **(E)** production of metabolites.

Meta-analyses have shown that some bacteria can directly promote lung cancer. For example, *HP* promotes the development of lung cancer 3.24 times faster than non-*HP* lung cancer patients (OR = 3.24, 95%CI = 1.11–9.47) (20), and *chlamydial* infection can increase the risk of lung cancer in men under 55 years of age (21). Also, *Mycobacterium tuberculosis (TB)* has a strong association with lung cancer, especially LUAD, while *TB* is also seen as a risk factor for non-smoking patients(RR = 1.8, 95%CI = 1.4-2.2 in non-smoking patients. RR = 2.9, 95%CI = 1.6-5.3 in smoking patients. RR = 1.6, 95%CI = 1.2-2.1 in LUAD patients) (22). In addition to direct carcinogenesis, some microbes can indirectly affect cancer, and the pathological mechanisms of microbial effects on lung cancer have been discussed in several studies (23, 24). Microbes have been found to exacerbate lung cancer progression through various processes, including altering host genes, activating oncogenic pathways, developing chronic inflammation, affecting the immune reaction, and generating metabolites (25). (i) Microbes can produce enzymatically active protein toxins that directly damage DNA or promote the production of reactive oxygen species (ROS) leading to DNA damage. For example, colibactin and cytolethal distending toxin (CDT) can lead to genomic destabilization and induce mutations (24, 26), while *Bacteroides fragilis* toxin (BFT) indirectly damages DNA by producing superoxide dismutase (SOD) (27). (ii) Microbial dysregulation creates an inflammatory cellular microenvironment that activates oncogenic pathways and exacerbates tumor progression (28). For example, *Veillonella* and *Prevotella* upregulate the expression of inflammatory mediators IL-1, IL-23, TNF, and IL-17, leading to the activation of ERK, PI-3K, and P53 signaling pathways, and promoting tumor cell proliferation (29, 30). (iii) Microbes bind to pattern recognition receptors (PRR), activating signaling pathways and inducing an inflammatory response. For example, toll-like receptors (TLRs) activate the cytokine Th17 by upregulating the NF-κB/STAT3 signaling pathway, which induces epithelial transformation and promotes tumorigenesis as well as host immune escape. Lipopolysaccharide from Gram-negative bacilli activates the TLR4/MYD88 innate immune signaling pathway leading to an increase in IL23 and IL17, which promotes an inflammatory response and exacerbates tumor progression (27, 30). (iv) Symbiotic microbes normally play an important role in both innate and adaptive immunity. In contrast, microbial dysregulation inhibits T and NK cell activity and promotes dendritic cell (DC), Treg, and M2 macrophage recruitment, resulting in host immune tolerance and tumor evasion of immune surveillance (23, 31). (v) Microbial metabolites are also involved in cancer metabolism. For example, bacterial metabolism can produce the carcinogen acetaldehyde. Furthermore, cyanotoxin production by cyanobacteria increases procyclic acidic repetitive protein 1 (PARP1) which enhances inflammation and promotes lung cancer (23, 30).In contrast, short-chain fatty acids (SCFA) and secondary bile acids (SBC) affect host immunity and promote the production of the anti-inflammatory factor IL-10, thereby reducing the incidence of cancer (32, 33). Thus, it appears that dysregulation of microbial homeostasis and reduction of commensal microbes affect multiple mechanisms of lung carcinogenesis. Still up for debate, however, is the exact causative link between microbes and lung cancer due to the intricacy of their interaction. More research into the processes is expected to pave the way for the prediction and dynamic monitoring of lung cancer (**Figure 1**).

## 4 Methods of detecting microbes

Earlier, the detection of microbes was limited to the culture of patient specimens. Bacteria can be identified at the level of cultured genera, which has the advantage of economy and isolation of viable bacteria, however, there are also disadvantages such as long detection times, fewer detectable species, and susceptibility to contamination (34). NGS technology further detects microbes in normal human lungs by gene amplification. Depending on the species detected, it is classified into 16S rRNA and18S rRNA. 16S rRNA, currently used for diagnosing bacteria and archaea, is the most commonly used and advanced technique (35). It sequences highly conserved sequences of microbes and subsequently performs operational taxonomic units (OTUs) classification (OTUs refer to microbes undergoing 16rRNA sequencing, and microbes that have 97% similarity in the rRNA sequences were classified and distinguished by a classifier algorithm (36)). Nevertheless, the results are limited to the phylum and genus level and do not provide a more specific detection (37). Whole-genome sequencing (WGS) allows for comprehensive classification and higher specificity of microbes by sequencing the genome with random primers. However, it has the disadvantage of being expensive and requiring the processing of large amounts of downstream data (38). Despite advances in microbial detection, sequencing tools are less sensitive to *TB* than traditional microbial cultures. Hence, combining sequencing with culture is a better assay that increases both the sensitivity of the assay and the abundance of functional microbes (39).

## 5 Sources of specimens of microbes

**Figure 2:**
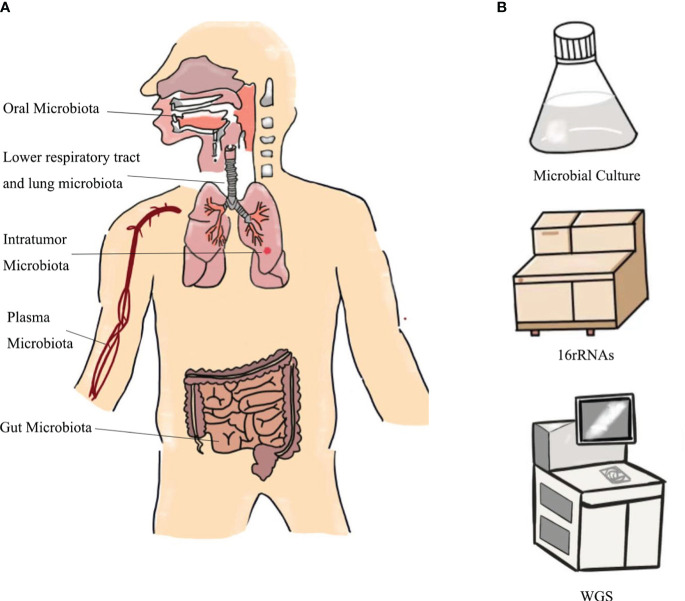
Sources of **(A)** microbial collection samples and **(B)** detection methods.

Microbiological detection of lung cancer has its unique advantages. This is because microbiological testing can be accomplished by collecting saliva, sputum, bronchoalveolar lavage fluid (BALF), bronchial epithelial brushings, and stool, which are noninvasive and convenient (**Figure 2**). However, different studies have shown different dominant genera in lung cancer patients, possibly due to interference from environmental factors, different collection methods and sample sources, and different selections of healthy controls (HC), etc. (10). We hypothesize whether a method could be devised to accurately control the type of sampling and reduce errors (25). We then explored the relationship between different sources of microbes and the diagnosis of lung cancer (**Table 2**). In addition, the differences between the microorganisms of different samples are demonstrated in **Table 3**.

**Table 2:** Summary of studies in this paper on microbes associated with the diagnosis of lung cancer.

Sample source	Sample types	Detection methods	Methods of analyses	Conclusions	Limitations
Oral cavity	Saliva (40)	16S rDNA V3 and V6	validated by qPCR	The combination of *Capnocytophaga* and *Veillonella* showed an ROC value of 0.86 with a sensitivity of 84.6% and a specificity of 86.7% in differentiating patients with SCLC from HC, and an ROC value of 0.80 with a sensitivity of 78.6% and a specificity of 80.0% in differentiating HC with LUAD.	The exact role of salivary bacteria in the development and progression of lung cancer is unknown
	Saliva and BALF (41)	Culturomics and 16S rRNA	Ns	*Streptococcus*, *Veillonella*, and *Prevotella* were enriched in oral samples, while *Pseudomonas* was enriched in BALF samples	Small sample size
Lower airways and lung	Sputum (42)	16SrRNA V3-V6	UniFrac and Mann-Whitney U test	Significantly increased abundance of *Haemophilus* and *Bergeyella* and a decreased abundance of *Atopobium* were found in lung cancer patients	A large number of patients are needed to confirm
	Sputum (43)	droplet digital PCR	Mann-Whitney U test and Logistic regression	*Acidovorax* distinguishes between SCLC and LUAD with an AUC of 0.86, sensitivity of 63.64%, and specificity of 96.30%. The combination of *Acidovorax* and *Veillonella* for the diagnosis of AUC of 0.89, sensitivity of 75.76%, and specificity of 88.61% for SCLC.	1. Small sample size 2. Sensitivity and specificity of biomarker detection is not enough
	BALF (44)	16S rRNA V1-V3	Mann-Whitney test, chi-square test, and Fisher’s exact test	*Veillonella* predicted lung cancer with an ROC value of 0.863 with a sensitivity of 65.0% and a specificity of 100.0%, while *Megasphaera* had an area under the curve of 0.781 with a sensitivity of 75.0% and a specificity of 25.0%. The combination of the two genera showed higher ROC values than alone AUC 0.888.	Further large-scale research is needed
	BALF (39)	WGS	Random Forest Regression	The abundance of lower respiratory tract microorganisms was reduced in lung cancer patients. The ability to identify cancer patients had AUCs of 0.882 and 0.796 in the training set and independent validation set, respectively.	1. Small sample size 2. An unclear causal relationship between microbes and lung cancer
	Epithelial brushing (45)	16S rDNA V4	Constructing a microbe-based classifier LMPC	LMPC predicted cancer incidence in both the test cohort and the validation cohort.	The subjects of this study were only a cohort of smokers
Tumor	Lung tissue (46)	RT-PCR	Ns	*Mycoplasma* strains were identified in all 32 patients	Additional studies are needed to further characterize the etiologic role of inflammation in lung carcinogenesis
	Lung tissue (47)	16S rRNA V4	Kruskal Wallis ANOVA and Wilcoxon rank sum test	The abundance of Proteobacteria (mainly *Acinetobacter* and *Acidobacterium*) was significantly lower in lung cancer patients and the prevalence of Phylum Firmicutes (*Streptococcus*) and Bacteroidetes (*Prevotella*) was higher.	1. The causal relationship between bacterial dysbiosis and disease is unclear 2. mNGS techniques were not used
Intestinal tract	Stool samples (48)	16S rRNA V3-V4	The VFDB database and the Kruskal-Wallis test.	The relative abundance of *Klebsiella* combined with that of *Streptococcus* yielded an AUC of 0.945, sensitivity of 0.882, and specificity of 0.919. The absolute abundance of *Haemophilus*, which can be used as a biomarker to differentiate between LC and NC, had an AUC of only 0.750, sensitivity of 0.941, and specificity of 0.625.	Further large-scale research is needed
	Blood and stool samples (49)	16S rDNA	LEfSe analysis and Wilcoxon rank sum tests	*Agathobacter* and *Blautia* are the main differential genera of early-stage NCLC. In addition, serum phospholipids and fatty acids are metabolites that regulate tumor cell growth, proliferation, and metastasis. They can be combined to diagnose lung cancer	1. The assay is based on 16rDNA only and is not as sensitive as mNGS 2. The specimens originated from cold regions where cold air exacerbates the progression of lung disease 3. Small sample size
	Stool samples (36)	16S rRNA	Support-Vector Machine (SVM)	An OTU-based predictor was developed with high accuracy in both the test cohort and validation cohort AUC of 97.6% and AUC of 76.4%, respectively.	1. 16S rRNA sequencing has limitations compared to mNGS 2. OTU markers do not distinguish smokers from non-smokers
Blood	Tissues and blood (50)	TCGA	Stochastic Gradient Boosting Machine (GBM)	Models can distinguish cancers based on microbial characteristics alone	1.ctDNA assay is plasma-based, not whole blood source 2. RNA data are not available, so it is not possible to assess whether mbDNA is from live or dead microorganisms
	human and microbial cfRNAs (51)	RNA-seq	SMART-based total RNA sequencing	The average recall was 52.5% using human cfRNA and 58.4% for microbial cfRNA profiles. When microbial and human cfRNA were combined, the AUC was 0.9 and the mean recall was 60.4%	Larger cohorts are still needed for clinical use.

**Table 3:** Current literature on comparisons of differences between oral microbes, lung microbes, gut microbes, and plasma microbes.

Microbes	Samples	Detection methods	Reference	Conclusions
Oral VS. Lung	Saliva VS. BALF	Culturomics and 16S rRNA	(41)	1. *Streptococcus, Veillonella*, and *Prevotella* were enriched in the oral samples, whereas *Pseudomonas* was enriched in the BALF samples 2. *Pseudomonas* and *Bacillus* were dominant in the LUAD samples. In contrast, in the LUSC samples, the dominant genera were *Pseudomonas* and *Streptococcus*
Lung	Sputum VS. BALF	16S rRNA	(52)	1. In BALF samples, *Firmicutes, Proteobacteria*, and *Prevotella* were the most predominant. In sputum samples, Firmicutes and Streptococcus were the most predominant. 2. Significant decrease in *Firmicutes* and genus *Streptococcus* in patients with distant metastases from LUAD, and a significant increase in genera *Veillonella* and *Rothia* in patients with distant metastases from LUSC
Lung	BALF VS. Lung tissue	WGS	(53)	1. Among NSCLC patients, *Streptococcus, Neisseria*, and *Enterobacter* dominated in BALF samples, whereas *Streptococcus*, *Enterobacter*, and *Mycobacterium* dominated in lobectomy samples 2. *TB* was significantly higher in lobectomy samples than in bronchoscopy samples
Oral VS. Lung	Saliva VS. Cancerous tissue VS. Paracancerous tissue	16S rRNA	(54)	1. *Promicromonosporacea* and *Chloroflexi* were increased in CT, while *Enterococcaceae* and *Enterococcus* were enriched in PT 2. At the genus level, *Pseudomonas*, *Bacteroides, Streptococcus*, and *Prevotella* were predominant in CT and PT. However, the dominant genera in saliva were *Prevotella, Streptococcus, Neisseria, and Veillonella*
Oral VS. Lung	Saliva VS. BALF VS. Cancerous tissue VS. Paracancerous tissue	16S rRNA	(55)	1. Higher predominance of *Streptococcus* and *Firmicutes* in the saliva of patients with NSCLC 2. Elevated levels of *Streptococcus, Prevotella*, and *Veillonella* (the three most abundant genera in saliva) were found in BALF compared to lung cancer tissue samples and they may be affected by microaspiration 3. *Proteobacteria* and *Firmicutes* were predominantly found in lung samples 4. No significant differences between these samples
Lung VS. Gut	Sputum VS. Fecal	16S rRNA	(56)	1. More diverse in sputum microbes and less diverse in gut microbes 2. Sputum microbiota performed the best in discriminating stage I to III patients from DM patients 3. Pseudomonas aeruginosa is detected in the sputum and feces of patients with brain metastases
Lung VS. Plasma	Cancer tissue VS. Blood	WGS	(57)	1. *Human pegivirus* (HPgV), *anelloviru*s, and *human endogenous retrovirus* (HERV) were detected in both blood and tissue samples, and a polyomavirus only in the cancer tissue samples 2. Compared with cancer tissue samples, the number of viral reads from *HPgV* and *anellovirus* in blood samples was much higher than those from cancer tissue samples

### 5.1 Oral microbes

The oral microbiome is second only to the gut microbiome in complexity, and it is also a source of microbiome for the lungs. When oral and exogenous microbiomes enter the lungs with respiration and become colonized, they may cause chronic infections and modulate host immunity (58, 59). Yan et al. were the first to identify that the saliva of lung cancer patients was significantly different from HC in the genera *Capnocytophaga* and *Veillonella* and suggested that these two microbes might be potential markers for the detection of lung cancer (40). Sun et al. also stated that *Streptococcus* and *Veillonella* were predominant in both their oral and lung samples (41). A meta-analysis in 2016 concluded that patients with periodontal conditions had a 1.26-fold increased risk of developing lung cancer in comparison to HC (HR = 1.24, 95%CI = 1.13-1.36) (60). These findings can demonstrate the potential viability of oral microbes as diagnostic markers for lung cancer.

However, the use of oral microbes alone as detection markers is susceptible to interference by smoking, environmental factors, and other chronic diseases of the host. Consequently, the researchers considered the combination of other components of saliva, such as DNA, mRNA, and proteins, with microbes for multi-omics analysis. Wei et al. developed an electric field-induced release and measurement (EFIRM) technique, which was used to identify EGFR mutations in body fluids. Their clinical study showed that EFIRM detected exon 19 deletion and exon 21L858R mutation with AUCs of 0.94 and 0.96, respectively (61). According to Zhang et al., a logistic regression model with an AUC value of 0.94 and a sensitivity of 82.81% that included five mRNA biomarkers (CCNI, EGFR, FGF19, FRS2, and GREB1) could distinguish between lung cancer patients and controls (62). Xiao et al. verified that the sensitivity of using protein levels such as haptoglobin, zinc-a-2-glycoprotein, and calreticulin for lung cancer differentiation was 88.5%, and the specificity was 92.3% (63). Liu et al. indicated that multi-omics analysis of saliva explains molecular interactions and facilitates causal inference. However, saliva’s standardized collection and analysis techniques need to be further improved (64).

### 5.2 Lower respiratory tract and lung microbes

In the past, healthy lungs were regarded as sterile. However, it has been shown that the lungs contain their microbiota since the development of high-throughput sequencing technologies. These microbes originate from the air, oral cavity, and upper respiratory tract and are influenced by bacterial migration, clearance, and replication. They can be regulated to replicate by local microenvironmental states or cleared by host coughing, ciliary motility, and innate and adaptive immunity, culminating in a dynamic equilibrium state in the body (65). Despite low lung microbial populations, they are critical for host inflammation and immunity (66). Lung microbes in healthy individuals are predominantly in the *Firmicutes* and *Bacteroides (* 67). Lung cancer, conversely, is associated with local microbial dysbiosis in the lungs, specifically an increase in the total microbiota and a decrease in diversity. Dong et al. demonstrated that *Veillonella* is significantly enriched in lung cancer and is the most strongly correlated potential marker for diagnosing lung cancer. *Prevotella* is also associated with lung cancer and acts synergistically with *Veillonella* can directly drive the PI3K/AKT (protein kinase B) and ERK/MAPK (extracellular signal-regulated kinase) signaling pathways, promoting lung cancer development (68, 69). *Veillonella* also promotes lung cancer development by inducing the recruitment of immune cells Th17 and neutrophils and by regulating tribes of microbiota, such as the aggregation of the pro-inflammatory bacterium *Pseudomonas aeruginosa (* 10). In contrast, the lower respiratory tract is more relevant to the staging and subtyping of lung microbes than the oral and upper respiratory tracts, making it an ideal substitute for lung cancer microbes during collection (52). Among the collection methods are sputum, bronchoalveolar lavage fluid, and lung tissue biopsy.

Sputum is a more representative sample than oral fluid as it is produced from the lower respiratory tract’s bronchi and tiny bronchioles. It may serve as a substitute for gathering lung bacteria collection when there is no oral microbial contamination. Druzhinin et al. used 16SrRNA for sputum microbiological detection. A significant increase in the abundance of *Haemophilus* and *Bergeyella* and a decrease in the abundance of *Atopobium* were found in lung cancer patients (42). Leng et al. investigated sputum microbes using droplet digital PCR and showed that *Acidovorax* and *Veillonella* have complementary roles. Their combination showed an AUC of 0.91, a sensitivity of 80%, and a specificity of 89.26% for the detection of squamous lung carcinoma. Additionally, *Acidovorax* also plays a function in distinguishing LUAD from LUSC with a sensitivity of 63.64% and a specificity of 96.30% (43). The investigators found that sputum microbes had a higher diagnostic sensitivity for LUSC. This may be because sputum is mainly secreted by the large airways and main bronchial tubes and LUSC also originates in the central lung tissue, whereas LUAD originates in the peripheral lung tissue (43).

Bronchoalveolar lavage examination is also a means of early detection due to the difference in microbiological composition between HC and lung cancer individuals. LEE et al. found that *Megasphaera* and *Veillonella* were more prevalent in lung cancer. When predicting lung cancer, *Veillonella* had an AUC of 0.863, sensitivity of 65.0%, and specificity of 100.0%, while *Megasphaera* had an AUC of 0.781, sensitivity of 75.0%, and specificity of 25.0%. Whereas the joint prediction of these two genera could show a higher AUC (0.888), their correlation needs further investigation (39). Subsequently, Marshall et al. constructed linear discriminant analysis (LDA) scores using the lung microbiome predictor of cancer (LMPC) to distinguish cancer status and predict cancer. They found that people with higher LDA scores had earlier onset of disease. However, this study focused only on the smoking cohort, and the absolute risk model was consistent with the incidence of cancer in ‘high-risk’ individuals. This suggests that LDA scores may be more applicable to high-risk individuals (45).

In addition, the finding that bacteria exist in tumors dates back more than a century. More in-depth studies were not carried out as contamination of tumor samples could not be ruled out (70). In 2011, Apostolou et al. found the presence of *Mycoplasma* in 32 resected lung cancer samples, and they proposed that chronic infection of the lungs is an important cause of tumorigenesis (46). Until 2020 Nejman et al. found that most tumors and surrounding normal tissues contained different types of bacteria and that these were predominantly identified in cancer and immune cells (71). Liu et al. subsequently found that the abundance of Proteobacteria (mainly *Acinetobacter* and *Acidobacterium*) was significantly lower in lung cancer patients by sequencing lung tissues and that the prevalence of Phylum Firmicutes (*Streptococcus*) and Bacteroidetes (*Prevotella*) was higher (47). Overall, sources of intratumoral microbes include microbial infiltration due to disruption of the mucosal barrier, microbial infiltration of adjacent tissues, and microbial transfer from the oral cavity and intestines (27). Intratumoral microbes can change the tumor microenvironment to affect the growth and metastasis of tumor cells. Still, the microbial abundance within the tumor is low and contamination during collection should be strictly avoided. In addition, the relationship between tumor and microbial action is unclear (active or passive? symbiotic or parasitic)?, and lung tissue biopsy specimens are not readily available. Researchers tend to use bronchoscopy or sputum specimens (70).

### 5.3 Gut microbes

Intestinal flora is a general term for the tribe of microbes in the gastrointestinal tract, accounting for 99% of the commensal bacteria in the human body. It aids in the breakdown and digestion of food, maintains normal physiological and immune functions of the intestinal tract, as well as the production of nutrients (25). Anaerobic bacteria are the main components of gut microbes and are also the main producers the leading producers of SCFA, butyric acid, in the human body. Possible reasons for some similarities between lung and gut microbes are: (i) The lungs and intestines are commensurate in embryonic origin, and the same mucous membrane covers them. (ii) Microbes inhaled by the mouth can enter the lungs and intestines. Bacteria from the digestive tract may crosstalk with respiratory microbes as a result of reflux (24). (iii) The channel through which the lung interacts with the intestine through blood vessels and lymphatics is known as the lung-gut axis (25). Through this axis, microbes that generate a local immunomodulatory response to the body also elicit a systemic response. In addition, ectopic intestinal flora and metabolites (such as SCFA) can elicit extraintestinal organ-specific immune responses (72). Due to the crosstalk between lung and intestinal microbes, it has been shown that gut microbial dysbiosis is highly related to lung disorders, including chronic obstructive pulmonary disease, asthma, pulmonary hypertension, and lung cancer (71).

The initial recommendation by Shen et al. was that lung cancer patients had higher absolute and relative abundances of *Haemophilus* and *Streptococcus*, which could serve as potential indicators for lung cancer detection. However, additional validation is required before these findings can be applied in clinical settings (48). Considering the complexity of microbial-host interactions and the fact that differences in microbes alone may not explain the role of microbes in cancer (73), Vernocchi, as well as Ni et al., proposed the use of combined microbe-metabolites for detection using multi-omics. Ni pointed out that *Agathobacter* and *Blaudia* are the main differential genera of early-stage NCLC. In contrast, metabolites such as serum phospholipids and fatty acids can regulate tumor cells’ growth, proliferation, and metastasis, and their combination with microbes could provide new ideas for lung cancer detection (49). Additionally, Zheng et al. used machine learning SVM to select 13 microbial markers based on OTUs for predicting the status and occurrence of diseases (36). However, Lim et al. indicated that fewer gut microbes were associated with lung cancer compared to oral microbes and that the gut microbes had limited predictive performance when machine learning was used to construct predictive models of non-smoking patient status (oral microbiome AUC 0.95 *vs*. gut microbiome AUC 0.76) (74). This may be related to oral microbes migrating to the lungs. Multiple researchers have also suggested that lower respiratory tract and lung microbes may be more associated with the development of lung cancer than gut microbes, which is more conducive to lung cancer diagnosis (37, 56).

### 5.4 Plasma microbes

Many microbes are present in healthy blood, and plasma microbial cell-free DNA (mcfDNA) may be diagnostic markers for liquid biopsies in lung cancer patients, according to recent studies (38, 75). In gastrointestinal tumors such as gastric, hepatic, and colorectal cancers, mcfDNA was found to have the ability to distinguish normal subjects from cancer patients (38). Subsequently, Poore et al. found that mcfDNA can also have good diagnostic ability in lung cancer through WGS combined with machine learning models (50). Zhou et al. showed a higher DNA abundance of *Selenomonas*, *Streptococcus*, and *Veillonella* in the circulating blood of lung cancer patients compared to HC, and the combined lung cancer detection of the three genera had a sensitivity of 75% and a specificity of 78% (76). Chen et al. proposed that the combination of human circulating free RNA (cfRNA) with the microbial cfRNA detection of lung cancer had an 8% increase in mean recall (the number of positive samples detected as a proportion of the actual number of positive samples) over human cfRNA detection alone (51).

In conclusion, the exploration of microbes has progressed from a high number of oral and gut microbes to a smaller and more refined number of intra-tumor microbes and plasma microbes. The goal of our study is to aspire to a balance between noninvasive and accurate detection.

## 6 Collection of microbes from different sources

### 6.1 Saliva

Subjects avoid brushing their teeth, eating, drinking, or chewing gum for at least 1 hour before saliva collection (77). Saliva collection is generally categorized as mouthwash, irritant saliva, and non-irritant saliva (78). Studies have shown that the microbial diversity of microbes collected by mouthwash does not differ from those collected by drooling (79). Still, mouthwash is more favorable for patients with dry mouths and takes less time (80). Since ethanol-free mouthwash is more favorable for sample transport, subjects were asked to rinse their mouths with 0.9% saline for approximately 30 seconds (81). For preservation, saliva samples should be centrifuged and the supernatant should be removed after collection at 4°C. The cellular precipitate is resuspended in sterile phosphate-buffered saline (PBS) and stored at -80°C. If cryogenic storage is not possible, the samples can also be made more stable by the addition of a preservative to make the cellular DNA more stable (82).

### 6.2 Sputum

When collecting sputum, subjects are required to blow their noses and rinse their mouths in advance as a means of minimizing the effects of postnasal drip and oral microbes (43). Typically, they are asked to cough up sputum spontaneously. When this is unsuccessful, participants are induced to undergo a 20-minute sputum nebulization with 3% saline solution (83), or sputum sampling using a lung flute (84). Sputum nebulization is time-consuming, and carries the risk of bronchospasm and respiratory infections, thus limiting its clinical use. The lung flute is a sound wave with a frequency of 18-22 Hz generated through exhalation, which enhances the mucus clearance system in the airways, making mucus secretions thinner and easier to expel through coughing (84). Sputum samples were collected in sterile containers and processed within 2 hours. Then, the specimens were selected microscopically by the investigator as a way to minimize contamination with oral squamous cells (43). Samples were treated on ice with 4 volumes of 0.1% dithiothreitol and 4 volumes of PBS, after which the samples were centrifuged and the supernatant removed. Finally, the remaining cell sediment was stored at -80°C until use (43, 85).

### 6.3 Bronchoalveolar lavage fluid

Subjects first received local anesthesia with lidocaine, followed by sedation with midazolam and fentanyl. Bronchial tubes were washed with 30-50 ml of sterile 0.9% physiological saline (39, 52), after which 3 ml of BALF was collected from each patient. The supernatant was removed by centrifugation at 4°C, 1 ml of DNA/RNA shielding was added, and the samples were finally stored in centrifuge tubes at -80°C (86).

### 6.4 Lung tissue

The most important aspect is to ensure proper sampling. Lung tissue was taken from the tumor, the area adjacent to the tumor, or an additional area away from the tumor (1-5 cm), as required. Surgically resected or punctured lung tissue samples were rapidly frozen in liquid nitrogen within 20 minutes and stored at -80°C until use (87).

### 6.5 Stool

The prerequisite for fecal collection is that subjects are asked to be free of antibiotic and glucocorticoid therapy within 3 months of specimen collection (88). Since the gut is mostly populated by anaerobic microbes, anaerobic fecal collection devices preserve as much microbial diversity as possible compared to conventional fecal collection devices (89). In addition, a fresh rectal swab can be used instead when the critical patient is unable to provide a fecal sample. However, the time to process the swab must be less than or equal to 2 days, after which time the quality of the sample will deteriorate rapidly (90). Stools and swabs should be frozen at -80°C immediately after collection and transported to the laboratory using dry ice (88). The fecal microbiome will remain stable at -80°C for up to two years with the addition of RNAlater, 95% ethanol, or preservation using FIT tubes and FOBT cards (91). If samples cannot be stored at -80°C, sample storage solutions such as Bgene-Gut, OMNIgene-Gut or RNAlater can be added to help preserve the microbiome as well (92).

### 6.6 Circulate blood

**Figure 3:**
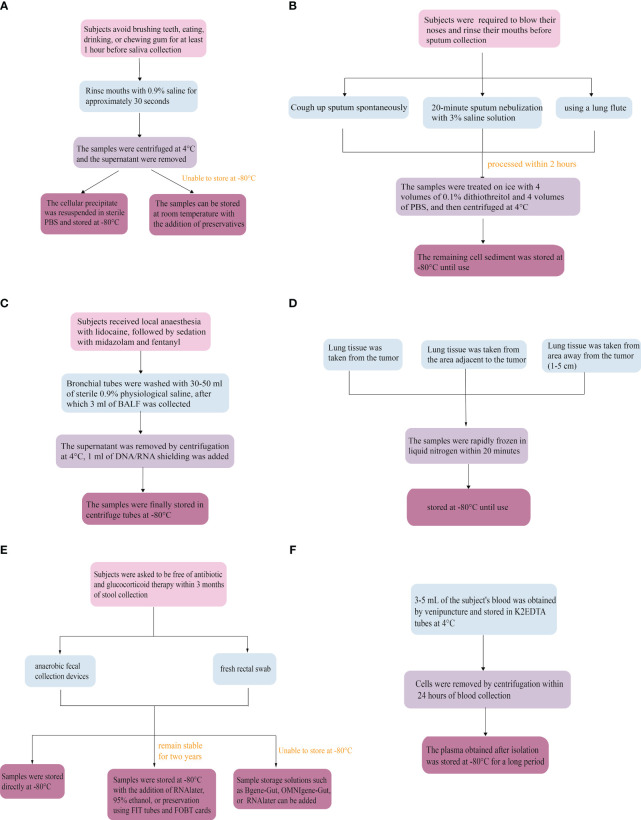
Microbiological collection methods. **(A)** Saliva, **(B)** Sputum, **(C)** Bronchoalveolar lavage fluid, **(D)** Lung tissue, **(E)** Stool, **(F)** Circulate blood.

3-5 mL of the subject’s blood was obtained by venipuncture through aseptic manipulation and stored in K2EDTA tubes at 4°C (93). Cells were removed by centrifugation within 24 hours of blood collection. Finally, the plasma obtained after isolation was stored at -80°C for a long period (93) (**Figure 3**).

## 7 Relationship between microbes and lung cancer typing and staging

Since microbes play an important role in the development of lung cancer by interacting with the host, we speculated whether microbes are heterogeneous and dynamically change on different pathological types and stages of lung cancer (94). Tasy et al. showed that there was a significant difference in β-diversity (diversity between different groups) between lower airway microbes of NSCLC and SCLC patients, which may be related to the different localization of the tumors. Furthermore, compared with stage I-IIIA NSCLC patients, the lower airways of stage IIIB-IV NSCLC patients were enriched with many oral commensals such as *Haemophilus*, *Fusobacterium*, *Gemella*, *Prevotella*, and *Granulicatella*. This may be due to the role of airway suction in causing microbial dysbiosis resulting in local inflammation and immunity, promoting NSCLC progression (95). In addition, Lu et al. demonstrated a significant increase in *Pseudomonas aeruginosa* abundance in sputum from NCLC patients with brain metastases (stage IV) by WGS (56). Yu et al. showed that *Thermus* was more abundant in patients with advanced lung cancer (stage IIIB-IV) and *Legionella* in patients with metastatic lung cancer (87). Further study by Huang et al. indicated that *Veillonella* and *Rothia* were increased in patients with LUSC metastasis (M1), whereas *Firmicutes* and *Streptococcus* were increased in LUAD distant metastasis. It may be because of microbial changes due to altered molecular biology during tumor metastasis (52). Li et al. combined microbes and mRNAs to construct a model for distinguishing lung cancer patients undergoing early (I) and intermediate to late (II-IV) stages using the Random Forest algorithm. The model had a prediction rate of 0.809 (96). From the above studies, we found that the dominant microbial species were different in patients with different histologic types and different stages. However, the veracity of the studies is limited by many factors such as the low incidence of SCLC, small sample size, and limited current studies. As well as most of the studies were only cross-sectional difference studies without dynamic monitoring (97). Therefore, microbes for typing and staging detection of lung cancer need to be further investigated.

## 8 Current problems with the use of microbes for the detection

### 8.1 Differences in individual samples

There is a complex symbiotic relationship between microbes and their hosts, leading to their influence by endogenous and exogenous factors in the human body. Endogenous factors include recombination, mutation, and amplification of genes, while exogenous factors include diet, sleep, exercise, chronic diseases, and drug use. Of these, antibiotics, chemotherapeutic drugs, and gastrointestinal infections have the greatest impact on microbes (98). How much these factors affect the composition of microbes and how we can analyze the data uniformly is open to further discussion (98).

Then, it is also well recognized that exposure to tobacco smoke increases the risk of lung cancer; nevertheless, a certain percentage of lung cancer cases also occur among non-smokers. Hence, some scholars also believe that non-smoking lung cancer belongs to “another type” of lung cancer (99). Smokers have reduced microbial diversity in the lungs, and the biology is enriched in the metabolic pathway of cigarettes (24). In contrast, non-smoking lung cancer patients have higher microbiological diversity, which may be related to chronic bacterial infections of the lungs (16). Therefore, their differences need to be considered when utilizing microbes for detection.

### 8.2 Differences in sample collection

Anaerobic bacteria dominate oral microbiology and intestinal microbiology, while aerobic bacteria dominate lower respiratory tract and lung microbiology. Therefore, different collection methods are required for different sources of microbes. In addition, when performing joint multi-omics analyses, the content and type of DNA, RNA, and proteins are also closely related to the collection method (64). For example, when collecting oral microbes, individual salivary glands have fewer non-salivary components, such as shed epithelium and food debris, making them more suitable for oral multi-omics analysis. In contrast, whole salivary glands are more suitable for functional analysis of saliva (64). We need to choose the most appropriate collection method according to different samples.

### 8.3 Limitations of microbes

A large number of studies and experiments have shown differences in the composition and number of microbes between tumor patients and normal subjects, and have explained that microbes are associated with lung cancer in a variety of ways. However, most of the clinical trials suffer from small sample sizes and researchers emphasize that there is no direct evidence of the exact role of microbes in carcinogenesis. Microbes are thought to promote cancer but not directly cause it (41). Sears et al. also stated that microbial detection is only valid when there is clinical suspicion of a tumor that current tests cannot diagnose. In the absence of confirmation of the location of the tumor, the test needs to be obtained from other specimens such as blood and urine (11). In addition, the role of less abundant microbes such as viruses and fungi in cancer has been neglected due to the limitations of the current level of microbial detection. The complex mechanisms of microbial action and causality in cancer have also not been investigated for the time being (37). Considering that there is no evidence of a direct role of microbes in cancer, it is not feasible to use differences between microbes from different patients to detect cancer alone. Therefore, it is necessary to combine other samples for testing (11).

## 9 Conclusion and discussion

Current diagnostic and screening tools for lung cancer are still limited. LDCT is radioactive and false-positive, while the biopsy is invasive and costly, so we need more convenient and accurate indicators. Many studies have shown that microbial responses to the host are bidirectional and that there are interactions between commensal microbes, host immune responses as well as lung cancer microecology (13). On the one hand, microbial dysbiosis promotes the development of cancer, and in animal experiments, antibiotic-administered mice are ineffective for radiotherapy and immunotherapy (100). On the other hand, we can artificially boost the beneficial bacteria to promote the efficacy of anti-tumor immunity and chemotherapeutic drugs and minimize immune-related adverse reactions (25). Additionally, we can restore the microbial ecology to support tumor treatment through dietary modification, oral probiotics, or fecal transplantation (101).In this paper, we summarize the mechanisms and methods of microbial lung cancer detection in recent years and further explore their feasibility and limitations. Firstly, we found that microbes can promote cancer progression by causing genetic damage to the host, affecting immunity, leading to chronic inflammation, and influencing metabolite. Second, lower respiratory tract microbes, lung microbes, and blood microbes can respond to the progression of cancer and are more favorable for early detection than oral microbes and intestinal microbes. Third, microbes can be detected in culture and non-culture. In the future, the combination of culture and sequencing (especially WGS) will be more accurate for detecting microbes in lung cancer. Moreover, some researchers have suggested that WGS needs to be based on a high microbial nucleic acid load basis. In contrast, macro-transcriptomics, macro-proteomics, and metabolomics can describe the downstream functions and metabolism of the active microbiota. Multi-omics analyses can be used to further explain microbe-host interactions (34, 102). Fourth, because of the complexity of the connection between microbes and their hosts, characterizing intergroup variations alone is insufficient to explain the function of microbes in different stages and types of lung cancer. Consequently, combining various biomarkers is required to perform precise diagnostic assessments, as well as multi-sample dynamic detection.

In addition, different experimental studies have yielded different species of dominant microbes in lung cancer patients, but there are some microbes and the effects they produce that have been repeatedly mentioned. These are precisely the focus of our subsequent studies (10). For example, *Veillonella* is potentially similar to *Prevotella* in up-regulating the ERK and PI3K signaling pathways to exert oncogenic effects (69). *Streptococcus* can induce chronic lung inflammation by upregulating pathways as well, but the exact role needs to be further determined. *Acidovorax* has a remodeling effect on the immune microenvironment of tumor cells, whereas *Haemophilus* and *Capnocytophaga* may be opportunistic pathogens, resulting from an impaired immune system.

Other biomarkers can be used in combination with microbes for detection. Liu et al. found that lung cancer patients grouped according to different serological markers also behaved differently regarding gut microbiology (103). Chen et al. used WGS to combine *Klebsiella, Mycobacterium, Pedobacter, Prevotella, Xanthomonas*, and NSE in alveolar lavage fluid for the detection of lung cancer, with an AUC value of 0.959, a specificity of 85.7% and a sensitivity of 100% (104). Various metabolites such as sphingomyelin and cysteinyl-valine were statistically different between lung cancer and normal samples (105). Chen et al. showed that L-valine and Lachnospiraceae_UCG-006 mutually regulate with each other through biological pathways (106). Ni et al. also indicated that *Agathobacter* and *Blaudia* are closely related to serum glycerophospholipid and sphingolipid lipid metabolism. They are all potential markers for the combined multi-omics detection of lung cancer (49).

Although microbes can be used as potential biomarkers for detection, there are many challenges in their practical use, with standardization being key to clinical use. It is important to note that the microbe is not used alone for lung cancer detection, but rather it is integrated into other markers of lung cancer for standardized procedural diagnosis. Future research will require longitudinal refinement of specimen collection methods, testing tools, and the incorporation of machine learning to build more accurate diagnostic models. For example, with the help of computers, efforts should be made to decontaminate, normalize data, and classify and analyze experimental specimens. Comparative analyses of oral, pulmonary, intestinal, and blood microbes and multi-omics analysis of microbes and gene-protein metabolites are needed for cross-sectional analysis. Finally, large-scale, multi-sample clinical validation is needed to perform. In addition, we also expect that the new methods can more accurately diagnose “harmful microbes” to provide clues for tumor staging and dynamic detection of microbial changes, which will allow for a dynamic view of the disease.

## Author contributions

SZ: Data curation, Writing – original draft. WZ: Software, Visualization, Writing – review & editing. HG: Data curation, Investigation, Validation, Writing – review & editing. YN: Formal analysis, Methodology, Project administration, Writing – review & editing. JS: Data curation, Project administration, Supervision, Validation, Writing – review & editing. PL: Funding acquisition, Resources, Writing – review & editing. YZ: Funding acquisition, Resources, Supervision, Writing – review & editing.
